# High-altitude adaptation in a flutter of sparrows

**DOI:** 10.1093/nsr/nwz175

**Published:** 2019-11-08

**Authors:** Yang Liu

**Affiliations:** 1 School of Life Sciences, Sun Yat-sen University, China; 2 Reviewer of NSR

Unraveling the genetic changes in adaptive evolution is a long-standing problem in biology [1]. Recent genome-wide studies provide unprecedented power to shed light on this fundamental question. High altitude represents life-threatening environmental conditions, such as hypobaric hypoxia, variable thermal regimes and strong ultraviolet radiation. As one of the thriving fields in evolutionary genomics, research on human [2] and wild organisms [3] at high altitude has contributed to our understanding on the genetic basis of such adaptations. In this issue of *National Science Review*, Qu *et al.* [4] provide an integrative exploration on phenotypic and genetic changes associated with a recent colonization in the Tibetan Plateau of an anthrodependent taxon: the Eurasian tree sparrow (*Passer montanus*).

Using population genomic approaches, the authors inferred that the highland sparrows colonized the Qinghai-Tibetan Plateau much less than 5000 years ago, broadly coinciding when barley/wheat agriculture was replacing millet. Rather than previous studies that focus on traits in response to hypoxia [2] and metabolism [3], the authors carried out comprehensive comparisons at muscle phenotypes and transcriptomic levels between highland and lowland sparrow populations. Their key finding reveals rapidly evolved muscle related traits are linked to changes in multiple loci in the highland population. The authors interpret this finding as polygenic adaptation from standing variation without causing the fixation of new mutations or large shifts of allele frequencies between populations. Their results are consistent with a growing body of evidence in human [5] and wildlife [6]. That is, standing genetic variation plays the predominant role in facilitating rapid adaptation.

This work represents a valuable effort that combines histology, experimental manipulation, transcriptomics and genomics to uncover high-altitude adaptation in birds. However, the authors acknowledge limitations in their acclimation experiment, which may have overlooked other factors contributing to plasticity in muscle phenotypes, e.g. developmental plasticity and epigenetic regulation. Efforts with more elaborate designs are hence needed to exclude alternative hypotheses. Nevertheless, together with another human commensal taxa, the House Sparrow (*Passer domesticus*) [7], the valuable genomic resources of these ‘small brown birds’ make them an emerging model system to understand how widespread organisms may thrive alongside humans.

***Conflict of interest statement***. None declared.
